# The first case of autochthonous subcutaneous dirofilariasis (*Dirofilaria repens*) in a dog from Białowieża (NE Poland) and possible threat posed to inhabitants of Białowieża Primeval Forest area

**DOI:** 10.1007/s00436-020-06955-2

**Published:** 2020-11-17

**Authors:** Marta Kołodziej-Sobocińska, Mariusz Miniuk, Małgorzata Tokarska

**Affiliations:** 1 Mammal Research Institute, Polish Academy of Sciences, Stoczek 1, 17-230 Białowieża, Poland; 2 Veterinary Office, Ogrodowa 4, 17-230 Białowieża, Poland

**Keywords:** Carnivores, Zoonosis, Wildlife, Domestic animals, Vector, Mosquito

## Abstract

We present the first case of dirofilariasis in a dog from Białowieża village located in the primeval European forest—Białowieża Primeval Forest (NE Poland). Molecular analysis of adult nematode specimens isolated from subcutaneous tissue confirmed the infection with *Dirofilaria repens*. An adult male dog has not travelled out of the Białowieża village for at least five years; therefore, we assume this is the autochthonous case of the disease. We discuss possible inter- and intra-species transmission routes of dirofilariasis on this territory, which is inhabited by diverse community of wild carnivores, domestic animals, and humans. We also discuss the likely sources of the disease in this, highly biodiverse unique European forest complex. We underline the lack of attention to this problem and its importance for veterinary, wildlife, and human health safety.

## Introduction

The ecology and epidemiology of helminths and arthropods of zoonotic potential shared by wild and domestic carnivores in Europe have been recently studied and reviewed (Otranto et al. 2015; Otranto and Deplazes 2019). Among zoonotic vector-borne nematodes, the genus *Dirofilaria* (Spirurida, Onchocercidae) is one of the best known and studied. This is mainly because of the worldwide distribution of the two main species, *Dirofilaria immitis* and *Dirofilaria repens*, the causative agents of canine heartworm and subcutaneous dirofilariasis, respectively (Otranto and Deplazes 2019; Genchi and Kramer 2020). Microfilaremic dogs are considered to be the most significant reservoir of dirofilariasis. In contrast to dogs, cats are not suitable hosts for these parasites and may develop only low-level, transient microfilaremia and are rarely positive for circulating microfilariae (McCall et al. 2008; Otranto et al. 2015; Genchi and Kramer 2017).

Canine subcutaneous dirofilariasis caused by *Dirofilaria repens* (Railliet and Henry, 1911) is one of the most common causes of human zoonotic dirofilariasis in the Old World (Colwell et al. 2011). Adult *D. repens* lives in the subcutaneous tissues of dogs and other carnivores, e.g. red fox *Vulpes vulpes*, grey wolf *Canis lupus*, raccoon *Procyon lotor*, where mature females release microfilariae into the blood of the infested hosts (Dantas-Torres and Otranto 2013). These microfilariae are ingested by several species of competent mosquito vectors during their blood-feeding (Cancrini et al. 2007; Capelli et al. 2018). *D. repens* has a long pre-patent period (up to 7 months) (Webber and Hawking 1955). Infective larvae migrate throughout the subcutaneous tissue and muscular connective fasciae, where they develop to the adult stage and reside permanently, even up to 4 years (Genchi and Kramer 2017). Often, the infection has no clinical symptoms. Occasionally, cutaneous disorders such as pruritus, dermal swelling, subcutaneous nodules containing the parasite, and/or ocular conjunctivitis can be observed (Albanese et al. 2013).

New endemic areas of *D. repens* occurrence have been identified and confirmed in several European countries, such as Austria, Czech Republic, Germany, Hungary, Poland, Russia, Ukraine, Slovakia, Turkey, and the Balkan Peninsula (Albania, Bosnia, Bulgaria, Croatia, Greece, Macedonia, Romania, Serbia), reviewed in Capelli et al. (2018). However, the occurrence of dirofilariasis in wildlife is still a very rarely studied and generally neglected health issue in Europe (i.e. Hurníková et al. 2012; Miterpáková et al. 2013; Ćirović et al. 2014; Ionica et al. 2017). It is noteworthy that in several eastern European countries *D. repens* prevalence is higher than *D. immitis* (Genchi and Kramer 2017).

The first cases of dirofilariasis in Poland were discovered in the central part of the country, canine dirofilariasis in 2009 (Demiaszkiewicz et al. 2009) and autochthonous human dirofilariasis in 2010 (the first case of human dirofilariasis in Poland was confirmed earlier—in 2007—but there was no evidence for its autochthonous origin) (Cielecka et al. 2012). Complex data of canine dirofilariasis in Poland was published in 2014 (Demiaszkiewicz et al. 2014). The authors examined over 1500 blood samples of dogs for microfilariae presence. *D. repens*–positive dogs were found in all 16 provinces of Poland; prevalence values differed from 1.2% in Małopolskie Voivodeship (southern Poland) up to 25.8% in Mazowieckie Voivodeship (central Poland) with the average of 11.7% for the whole country (Demiaszkiewicz et al. 2014). Adult stages of *D. repens* have been rarely reported in dogs in Poland, only few cases of the parasite located in the cave on scrotum, subcutaneous connective tissue, in a nodule on the eyelid, and inside a cyst in the testicular parenchyma (Demiaszkiewicz 2014).

We describe the first autochthonous case of canine subcutaneous dirofilariasis in an adult male dog inhabiting Białowieża village, located in the Białowieża Primeval Forest (BPF), partially protected as Białowieża National Park (NE Poland, Podlaskie Voivodeship). It is the first molecularly confirmed report of *D. repens* occurrence on this territory. BPF offers a broad spectrum of possible wild canid and felid hosts inhabiting BPF (among others—grey wolf, red fox, Eurasian lynx), while lack of information on possible *Dirofilaria* arthropod vectors and no diagnosed cases of dirofilariasis in dogs and cats inhabiting forest villages creates the ground for further research on transmission routes of *D. repens* between wild, domestic canids and humans in a model forest ecosystem which is BPF with its unique biodiversity.

## Material and methods

### Case description

**Fig. 1:**
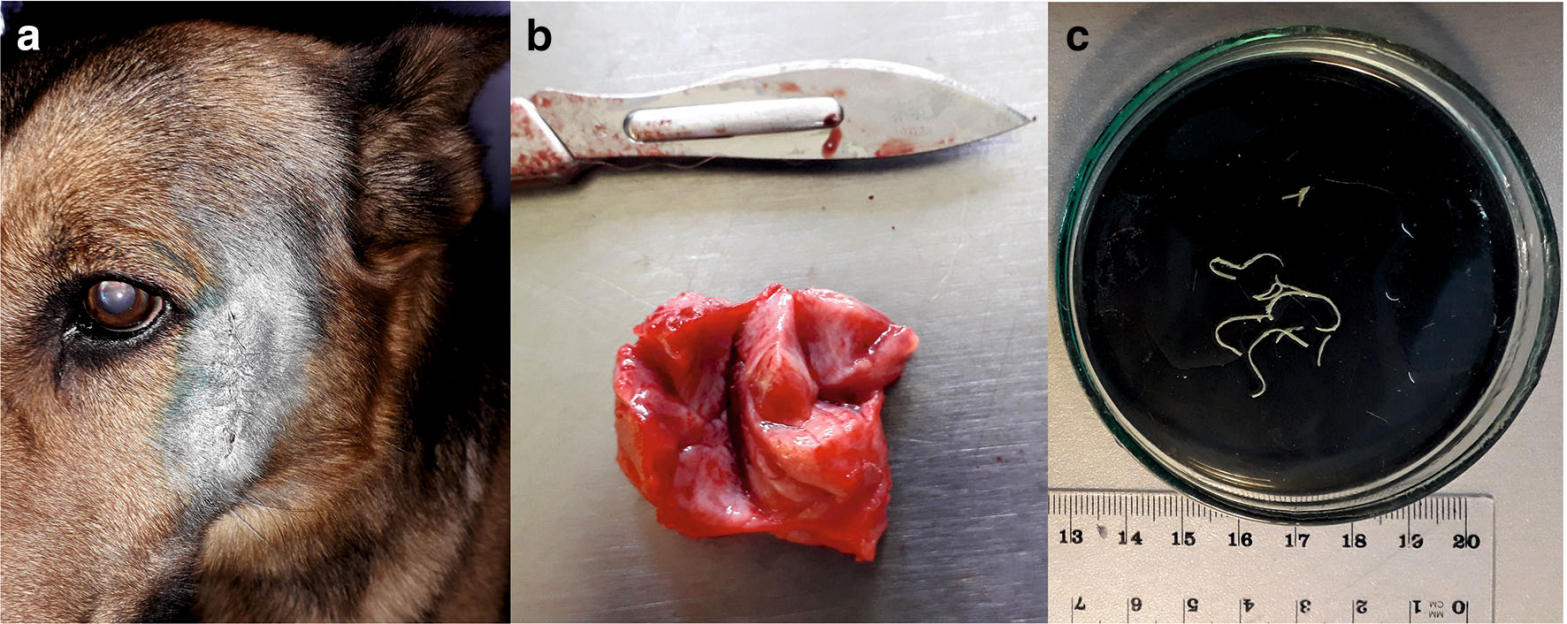
A case of subcutaneous dirofilariasis in a dog from Białowieża village. (**a**) The location of the scar after removal of the nodule containing *Dirofilaria repens*; (**b**) surgically removed nodule; (**c**) *D. repens* specimens extracted from the nodule. Photo: Marta Kołodziej-Sobocińska

Nodule-type skin lesion was found in a ten-year-old male dog, German Shepherd crossbreed (35 kg), from Białowieża. The dog was adopted from a local shelter and has not left Białowieża since 2015. The nodule was located in the subcutaneous tissue of the cheek near the external angle of the left eye (Fig. 1A). A fast-growing lesion reached a diameter of 2 × 2 cm within 2 weeks from the moment the first skin changes have been observed (Fig. 1B). The nodule was removed surgically in August 2019. A cluster of several nematodes (3 specimens) was found in the cross-section (Fig. 1C). Monthly administration of the medicine containing imidacloprid and moxidectin was prescribed for 6 months.

### Genetical analysis

The three isolated nematodes were preserved in 99% alcohol. DNA from all the 3 samples have been extracted using Sherlock AX (A&A Biotechnology). Two of them have been preliminarily crushed in mortar and then incubated overnight in the buffer, while the remaining one was, alternatively, frozen/heated several times, then crushed in mortar and subjected to DNA extraction using Sherlock AX. We obtained 10× more DNA with the freezing/heating option.

**Fig. 2:**
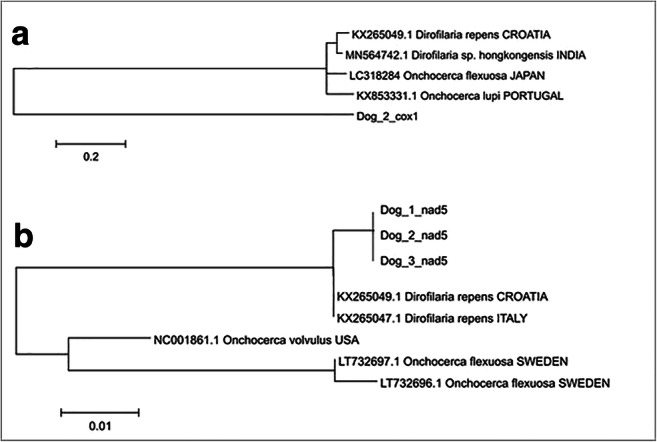
The phylogeny of the DNA sequences obtained in the study and reference sequences of *Dirofilaria* sp. and *Onchocerca* sp. from GenBank. (a) cox1. (**b**) nad5

**Fig. 3:**
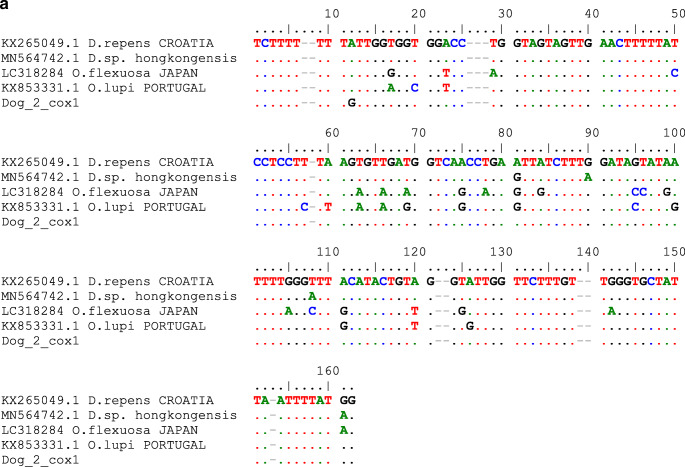

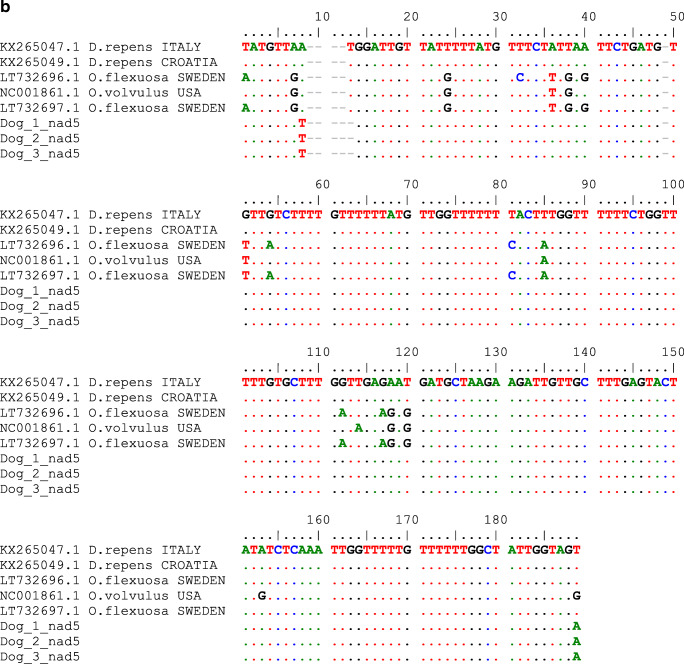
(**a**) Cox1 mitochondrial gene sequence alignment for *Dirofilaria repens* achieved in this study and GenBank sequences of *Dirofilaria* sp. from India (MN564742.1), *Dirofilaria repens* (KX265049), and closely related *Onchocerca lupi* (KX853331). (**b**) nad5 mitochondrial gene sequence alignment for *Dirofilaria repens* achieved in this study and GenBank sequences of *Dirofilaria repens* (KX265049, KX265047) and closely related *Onchocerca volvulus* (NC001861) and *Onchocerca flexuosa* (LT732696). Dots instead of letters indicate sequence identity with the reference (bold) sequence

Two mitochondrial regions were sequenced and analysed: cytochrome c oxidase subunit 1 gene (cox1) and subunit of the respiratory NADH dehydrogenase gene (nad5). We used starters designed for *Onchocerca* genus described by Ilhan et al. (2013): D&Ocox1F: 5′-GCT TTG TCT TTT TGG TTT ACT TTT G-3′ and D&Ocox1R 5′-CCA TAA AAT TAA TAG CAC CCA AC-3′; D&Onad5F: 5′-CCT GTT AGT TGT TTG GTT CAT AGT AG-3′ and D&Onad5R: 5′-CTA CCA ATA GCC AAA AAA CAA AAA CC-3′. The obtained sequences were analysed using online BLAST tool (https://blast.ncbi.nlm.nih.gov/Blast.cgi) and used for phylogenetical studies. We aligned the newly achieved sequences with adequate cox1 and nad5 gene fragments of selected *Onchocerca* species available at GenBank (Figs. 2 and 3). The phylogenetic trees were created using the maximum likelihood method and Tamura-Nei model (Tamura and Nei 1993). The trees with the highest log likelihood (− 1811.37 for cox1 and − 303.42 for nad5) are shown. Initial trees for the heuristic search were obtained automatically by applying Neighbor-Join and BioNJ algorithms to a matrix of pairwise distances, estimated using the Tamura-Nei model, and then selecting the topology with superior log likelihood value. The trees are drawn to scale, with branch lengths measured in the number of substitutions per site. This analysis involved 5 (cox1) and 11 (nad5) nucleotide sequences. There was a total of 178 (cox1) and 184 positions in the final dataset. Evolutionary analyses were conducted in MEGA X (Kumar et al. 2018).

## Results

A nodule of approximately 2 by 2 cm in size was removed during a surgery (Fig. 1B). Three nematode specimens 3–5 cm long were found and extracted from the lesion (Fig. 1C). Nematodes were accidentally damaged during the surgery; therefore, morphological evaluation was impossible. Thus, genetic analyses were performed to confirm the parasite species. The obtained DNA sequences showed 99.45% identity with *Dirofilaria repens* nad5 reference sequences and 98 to 100% identity with *Dirofilaria repens* cox1 reference sequences in GenBank (Figs. 2 and 3). Figure 2A and Fig. 2B show the results of evolutionary analyses of both mitochondrial regions. The cox1 gene analysis did not clearly indicate *Dirofilaria* genus as the evolutionary closest, but NADH 5 sequences supported the identification of the analysed helminths as *Dirofilaria repens*. To exclude possibility of unintentional amplification of a pseudogene, we translated the obtained NAD5 sequences and performed Protein Blast that ensured us that the conclusion of our finding is correct. The sequences used for evolutionary analyses are presented in Fig. 3. Obtained sequences were deposited in GenBank, accession nos. MT671363 to MT671368.

## Discussion

Our study reports the first case of subcutaneous dirofilariasis caused by *Dirofilaria repens* in a dog from Białowieża village. The village is located in the middle of the BPF. However, there is no data about *D. repens* occurrence in wild carnivores from BPF. Potentially, BPF is a suitable place for the spread of dirofilariasis, because it is inhabited by diverse community of 12 species of carnivores (potential hosts for *D. repens*), such as canids—grey wolf *Canis lupus*, raccoon dog *Nyctereutes procyonoides*, red fox *Vulpes vulpes*; felids—Eurasian *Lynx Lynx lynx*; and mustelids—European badger *Meles meles*, European polecat *Mustela putorius*, pine marten *Martes martes*, stone marten *Martes foina*, river otter *Lutra lutra*, American mink *Neovison vison*, least weasel *Mustela nivalis*, and ermine *Mustela ermine* (Jędrzejewska and Jędrzejewski 1998). Thus, possible inter- and intra-species transmission between dogs and wild carnivores, as well as humans inhabiting this territory, cannot be neglected. *D. repens* in wildlife is found rarely; in Europe, subcutaneously located adult forms of *D. repens* were diagnosed in two wolves and a red fox from central Balkan—Macedonia, Serbia (Ćirović et al. 2014). Moreover, DNA of *D. repens* has been searched in multispecies study conducted partially on the same species as those inhabiting BPF (except American mink) in Romania (Ionica et al. 2017); the parasite DNA was found in two red foxes, one grey wolf, and one least weasel; total prevalence among studied carnivores was 1.4% (Ionica et al. 2017). *D. repens* occurrence was also proved by PCR in stone marten and red fox in Slovakia (Hurníková et al. 2012; Miterpáková et al. 2013) and in red fox from Italy (Magi et al. 2008). These findings suggest a potential role of wild carnivores in the epidemiology of the disease. The rarity of dirofilariasis detection in wildlife and moreover, in protected areas, is related to the fact that the evidence of adult *D. repens* is possible only at necropsy (in the subcutaneous tissue) and it is more difficult than *D. immitis* (in the pulmonary arteries and in the heart); thus, there are only fragmentary data about the distribution of this nematode in wild carnivores (Otranto and Deplazes 2019).

Mosquitoes—as vectors—are necessary for the spread of dirofilariasis. Available information on mosquitoes inhabiting BPF is scarce and outdated (Skierska 1959; Wegner 1999); thus, the knowledge about the potential of arthropod vectors for *D. repens* transmission on studied territory is missing. Xenomonitoring–the detection of the parasite in blood-feeding arthropods—is known as a useful tool for the assessment of the *D. repens*–infected vertebrate hosts (Masny et al. 2016). Thus, confirmed here the first case of *D. repens* in BPF opens the field for further research. Moreover, it is the only one of just few cases of canine subcutaneous dirofilariasis in Poland (see Demiaszkiewicz 2014) which was undeniably confirmed genetically as caused by *D. repens*.

We assume that the reported case of canine dirofilariasis is autochthonous, as the infected dog has not travelled outside the village since 2015. Before, it travelled only twice to Warsaw in December, when the possibility of being bitten by mosquitoes is almost non-existent. Thus, the dog had to be infected by a mosquito vector in BPF. Another question is how has dirofilariasis emerged in this area? Whether it was a natural spread of the disease, which is observed with the expansion of mosquito vectors due to climate warming (Otranto and Deplazes 2019), or the disease was brought in by tourists and their microfilaremic dogs. Microfilaremic dogs are thought to be the most important reservoir for *D. repens* infection (Capelli et al. 2018). In both cases, more favourable environmental and climate conditions associated with global warming allowed the nematode to survive in mosquito vectors and, as a result, transfer the infection to the local dog. We also do not know if it is only an accidental case or *D. repens* (and maybe also *D. immitis*) or it is already circulating in the environment of BPF. Only further studies may resolve this conundrum. They should be carried out in three directions: (1) veterinary—screening for microfilariae occurrence in blood of dogs and more attention given to surgically removed subcutaneous nodules which may contain adult *D. repens*; (2) wildlife study—searching for adult parasites in dissected carnivore carcasses and/or for *D. repens* DNA in tissue or organs (e.g. spleen; see Ionica et al. 2017); (3) complex xenomonitoring of mosquito vectors.

This first record of canine subcutaneous dirofilariasis in BPF incites us to discuss the need for more extensive research of this disease, not only in domestic animals and humans but also in the wildlife. The consequences of the pathogens expansion into the protected ecosystems have not been studied, especially in the context of climate change favouring new disease occurrences. Highly biodiverse ecosystems may be crucial for the effective pathogen spread. We propose and encourage governments, scientists, veterinarians, and other entities to put more emphasis on the potential impact of emerging diseases on vulnerable wild host species inhabiting nature protected areas as well as on people and domestic animals living in their neighbourhood. Dirofilariasis appears to be a serious disease for both animals and humans, but limited knowledge on its etiology restricts the possibility of the comprehensive evaluation of its occurrence and spread in both wild and urban areas.
